# Health-related quality of life in family members of patients with an advanced cancer diagnosis: A one-year prospective study

**DOI:** 10.1186/1477-7525-10-89

**Published:** 2012-07-30

**Authors:** Catarina Sjolander, Bo Rolander, Johannes Järhult, Jan Mårtensson, Gerd Ahlstrom

**Affiliations:** 1 The School of Health Sciences, Jönköping University, P.O. Box 1026, SE-551 11, Jönköping, Sweden; 2 The Ryhov County Hospital, SE-551 85, Jönköping, Sweden; 3 The Swedish Institute for Health Sciences, Department of Health Sciences, Lund University, Box 187SE–221 00, Lund, Sweden

**Keywords:** Family member, Advanced cancer, Health-related quality of life, Mental health dimension, Physical health dimension

## Abstract

**Background:**

Receiving a cancer diagnosis affects family members as well as the person diagnosed. Family members often provide support for the sick person in daily life out of duty and love, and may not always think of their own vulnerability to illness. To individualise support for them, family members who are most at risk for becoming ill must be identified.

The aim of this study was to investigate health-related quality of life (HRQOL) in family members of patients with advanced lung or gastrointestinal cancer 3 to 15 months after diagnosis.

**Methods:**

Data on mental and physical dimensions of HRQOL were collected from family members of these patients in this prospective quantitative study. Five assessments using the Short Form 36 Health Survey (SF-36) and EuroQol (EQ-5D) were conducted during a 1-year period starting 3 months after diagnosis. Thirty-six family members completed the study, i.e. participated in all five data collections.

**Results:**

No statistically significant changes in physical or mental HRQOL within the study group appeared over the 1-year follow-up. Compared with norm-based scores, family members had significantly poorer mental HRQOL scores throughout the year as measured by the SF-36. Family members also scored statistically significantly worse on the EQ-5D VAS in all five assessments compared to the norm-based score. Findings showed that older family members and partners were at higher risk for decreased physical HRQOL throughout the 1-year period, and younger family members were at higher risk for poorer mental HRQOL.

**Conclusions:**

It is well known that ill health is associated with poor HRQOL. By identifying family members with poor HRQOL, those at risk of ill health can be identified and supported. Future large-scale research that verifies our findings is needed before making recommendations for individualised support and creating interventions best tailored to family members at risk for illness.

## Background

The diagnosis of malignant disease causes serious psychological distress to the affected person, and the illness can have severe consequences for the family [1-4]. Family members often spend a lot of time providing the diseased person with practical and emotional support during, between and after treatments. Supportive care from family members, in addition to emotional support, includes different kinds of assistance, such as help with household tasks, transportation, medical appointments and medication management [5,6].

Previous studies on family caregivers of persons with advanced cancer have shown that their mental health is negatively affected [7,8]. They are at high risk of becoming anxious and depressed in response to the critical situation [1,9]. Grunfeld and colleagues [7] have in fact reported that significantly more family caregivers than patients are anxious [7]. When health-related quality of life (HRQOL) has been evaluated in patients’ family members, results have been inconsistent. That is, some studies have shown HRQOL to be negatively affected by the situation [8,10-12], whereas others have reported family members’ mental and physical health comparable to that of the general population [13].

In a review of the literature on effects of caring for a patient with cancer, emotional and social concerns were the most identified types of family caregiver problems [14]. However, there are some knowledge gaps in studies on specific problems and burdens associated with a patient’s cancer diagnosis. Less focus in the literature has been on physical health, with the most prevalent problems reported being pain, sleep disturbances, fatigue, loss of appetite and weight loss [15,16]. However, only a few studies have addressed how caregiver problems change during different stages in a cancer patient’s illness trajectory [14]. Therefore, it is important to learn more about the start of the cancer trajectory and how it is experienced over time from a family member’s perspective related to changes and existential threat [17-20].

Earlier studies on family members’ HRQOL have been conducted during palliative care starting from the cancer patient’s diagnosis, with the centre of attention at the time point close to or after death [11,21].

Those diagnosed with lung or gastrointestinal cancer have a poor prognosis and low survival rate regardless of treatment or differences in progression of the disease. In fact, lung cancer, followed by stomach and liver cancer, are the most common causes of cancer deaths worldwide [22]. The poor prognosis associated with these forms of cancer can mean extra pressure on relatives and may affect family members’ HRQOL. Therefore, the aim of this study was to investigate HRQOL in family members of patients with advanced lung or gastrointestinal cancer over a 1-year period. There is a need to identify family members who are most at risk for becoming ill to determine if there is a need for tailored support for the next of kin. The term “advanced cancer” in this study is based on a clinical perspective meaning a severe form of cancer with a high rate of mortality.

## Methods

### Design

This was a prospective quantitative study designed to follow mental and physical dimensions of HRQOL in family members of persons with advanced lung or gastrointestinal cancer over a 1-year period, beginning 3 months after diagnosis. The study was conducted during 2007–2011. Data were collected on five different occasions, 3, 6, 9, 12 and 15 months after the patient’s diagnosis, and are termed assessments A1, A2, A3, A4 and A5, respectively.

### Research ethics

Ethical approval for this study was granted by the research ethics committee at Linköping University, Sweden. Informed written consent was obtained from all participants prior to the study. It was made clear to them that participation was voluntary, and they were free to withdraw whenever they wished without any consequences related to care for themselves or the cancer patient. Confidentiality was guaranteed, and the findings could not be linked to the individuals.

### Selection of the study group

Family members incl`ded in this study had a sick relative who up to 3 months earlier had been diagnosed with advanced lung cancer or cancer in the upper gastrointestinal tract at either one medical clinic or one of the two surgical clinics at two hospitals in the south of Sweden. Eligible family members had to be aged 18 years of age or older and able to speak Swedish.

Thirty-six family members completed the study, i.e. participated in all five data collections. The mean age of the study group was 63 years (SD = 16 years). The family members in the study group were next of kin to a patient with lung cancer (n = 24, 66%) or a patient with gastrointestinal cancer (n = 12, 34%). Gastrointestinal cancer in this study included pancreatic, oesophageal, liver and stomach cancer. In both lung and gastrointestinal cancer, there are several different cancer diagnoses that lead to different courses of the diseases, but all involve a poor prognosis. Participant characteristics, including gender, education, work status and relationship to the patient with cancer, for the study group and the 21 individuals who dropped out after the study began are presented in Table 1.

**Table 1:** **Characteristics of the study group (n = 36**) **and those who dropped out after the study began (n = 21**)

	**Study group¤**	**Drop-outs**
	**n (%)**	**n (%)**
**Gender**		
Female	26 (72)	13 (62)
Male	10 (28)	8 (38)
**Education**		
High school and above	24 (66)	16 (76)
Less then high school	12 (33)	5 (24)
**Work status**		
Retired	19 (52)	13 (62)
Currently working	14 (39)	7 (33)
On sick leave from work	3 (9)#	1 (5)#
Applying for a job		
**Relationship to the person with cancer**		
Partner	26 (72)	16 (76)
Grown child	8 (22)	4 (19)
Other relative (ex-partner or sibling)	2 (6)#	1 (5)#
Primary caregiver	31 (86)	18 (86)
Not primary caregiver	5 (14)#	3 (14)#

¤The study group participated in all five measurements. Drop-outs participated in fewer than five measurements.

There was no significant difference in characteristics between the study group and drop-outs examined with the chi-square test.

#Not tested for a difference between the study group and dropouts.

**Figure 1:**
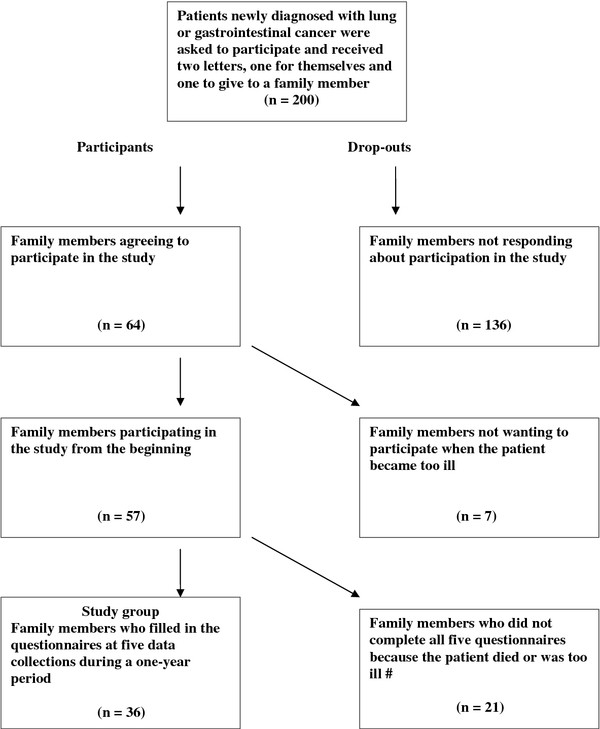
**Flow chart of patients and family members during the study.** Note: # Completed questionnaires in the drop-out group varied between one and four per person, with a total of 51.

Initially, 200 patients with cancer were asked by one of eleven nurses or one of two physicians at the three clinics if they were willing to give written information about the study to their closest family member and ask them to participate (Figure 1). If patients accepted, they received two letters indicating the purpose and design of the study, one to themselves and one to the chosen family member. Sixty-four family members agreed to participate, but seven of them withdrew before the study began because the patient was too ill. Demographic data on those who withdrew prior to the start of the study (n = 7) were not available. Twenty-one family members dropped out because the patient died or became too ill. Two types of calculation were used to analyse differences between the study group and drop-outs. First, any significant differences in demographic data (age, gender, education, occupation or relationship) were examined (Table 1). Second, differences in HRQOL between the groups were explored at baseline. No significant differences were found between the study group (n = 36) and drop-outs (n = 21) in either calculation.

### Data collection procedure

Family members who gave their written consent to participate were contacted by phone by the first author. They were then mailed two HRQOL self-report instruments together with a cover letter every 3rd month. To reduce the number of drop-outs, they were also phoned 1 week before each data collection to remind them that the questionnaires were going to be mailed again. A similar reminder was provided 2 weeks after the mailed questionnaires if their response had not reached the first author.

### Instruments

The two self-report HRQOL instruments used in this study were the Short Form Health Survey (SF-36) and the EuroQol (EQ-5D). These instruments were selected because they are considered complementary in respect to different variables of similar dimensions.

### Health-related quality of life (HRQOL)

The SF-36 is one of the most widely used self-assessment instruments for measuring HRQOL. It consists of 36 items making up eight scales that measure a physical and a mental health dimension. The physical health dimension, represented by the Physical Component Summary Score (PCS), contains the scales Physical Functioning (PF), Role Physical (role limitations due to physical problems; RP), Bodily Pain (BP) and General Health (GH). The mental health dimension, represented by the Mental Component Summary Score (MCS), contains the scales Vitality (VT), Social Functioning (SF), Role Emotional (limitations due to emotional problems; RE) and Mental Health (MH) [23]. Raw scores were coded, re-calibrated, summated and transformed from 0 to 100 for each item, with higher scores reflecting better HRQOL, following norm values for the general Swedish population. The eight scales and the two dimensions, PCS and MCS, were calculated according to the standard SF-36 algorithms in the test manual [23,24].

The EQ-5D, also a self-assessment instrument, comprises five dimensions: mobility, self-care, usual activities, pain/discomfort and anxiety/depression. The answers were scored by an algorithm from the EQ-5D manual [25,26], an index value of HRQOL with higher scores reflecting better health status. The respondents were also asked to draw a line on a visual analogue scale (VAS) thermometer that ranged from the worst imaginable health state today (0 points) to the best imaginable (100 points). The index value of the EQ-5D can be generated from the dimensions with a range of −0.594 to 1 by applying scores from standard population values [27].

### Analysis

Family members’ characteristics in the drop-out group compared with the study group were examined using the chi-square test. Linear mixed models with repeated observations and method restricted maximum likelihood were applied to analyse changes between 3 months and 15 months for family members in separate comparison analysis of each value, with measure 1 at baseline (3 months after the patient’s diagnosis). The variable of age was dichotomised on equal percentiles in two age groups (20–65 and 66–84 years). Because the study group was small, two equally sized groups were preferable for statistical comparisons. Moreover, such classification meant that the older group came to consist mainly of retired individuals, and the younger group comprised mainly people who were economically active. SF-36 dimensions were analysed with the Mann–Whitney *U* Test for independent samples between age group and between family members’ relationship to the patient. Although the analysis was non-parametric, the family members’ HRQOL on the SF-36 and EQ-5D are presented in the tables as means, medians and standard errors of the means to facilitate comparisons to previous studies. The associations between age and relationship to the patient with cancer (partner or grown child) were calculated using Spearman’s correlation coefficient. A stepwise logistic regression analysis using the forward Wald method was conducted on all five assessments with the eight scales in the SF-36 as independent variables and age and relationship as dependent variables. The aim was to describe which scales had the greatest effect by age and relationship. The significance level was assumed at α = 0.05, and the statistical calculations were carried out in SPSS, version 19.1. Statistical differences were tested between norm-based scores for the SF-36 and EQ-5D and respective mean scores at A1–A5 using Statistica, version 10. Because age may be an important factor in HRQOL, it was important to get a comparable age structure in a comparison of scores of the study group and norm-based scores.

## Results

Table 2 shows results for physical and mental HRQOL measured by the SF-36 and EQ-5D on 5 different occasions over 1 year, beginning 3 months after the cancer diagnosis. There were no statistically significant differences in HRQOL within the study group over the 15-month follow-up.

**Table 2:** HRQOL scores of family members at five assessments compared to norm-based scores (n = 36)

	**Norm-based score □ # M [SE]**	**A1 Md**	**A1 M [SE]**	**A2 Md**	**A2 M [SE]**	**A2-A1 p-value**	**A3 Md**	**A3 M [SE]**	**A3-A1 p-value**	**A4 Md**	**A4 M [SE]**	**A4-A1 p-value**	**A5 Md**	**A5 M [SE]**	**A5-A1 p-value**
PCS	□44.6 [±1.02]	45	46 [±1.9]	52	49 [±2.0]*	0.33	48	46 [±1.8]	0.96	48	45 [±2.0]	0.66	51	47 [±2.0]	0.66
MCS	□51.1 [±0.97]	38	39 [±2.2]*	44	42 [±2.5]*	0.53	38	39 [±2.4]*	0.91	45	43 [±2.2]*	0.25	45	41 [±2.4]*	0.71
PF	□78.2 [±1.94]	83	79 [±3.6]	90	83 [±3.2]	0.42	89	80 [±3.2]	0.77	85	80 [±3.4]	0.85	90	81 [±3.6]	0.69
RP	□70.1[±3.42]	75	63 [±6.4]	100	74 [±6.4]	0.22	88	60 [±7.7]	0.77	75	62 [±7.2]	0.89	88	66 [±7.1]	0.76
BP	□66.0 [±2.35]	56	63 [±4.8]	62	67 [±4.4]	0.55	52	63 [±4.2]	0.95	51	60 [±4.9]	0.67	62	64 [±4.2]	0.82
GH	□67.2 [±2.04]	59	60 [±3.4]	67	64 [±3.1]	0.33	65	62 [±3.1]	0.65	67	62 [±3.5]	0.63	67	65 [±3.6]	0.28
VT	□67.3 [±2.09]	48	50 [±3.3]*	58	57 [±3.9] *	0.19	50	54 [±3.9]*	0.47	58	55 [±4.0]*	0.39	58	55 [±3.7]*	0.36
SF	□88.3 [±1.73]	75	74 [±4.3]*	94	79 [±4.2] *	0.45	69	70 [±4.3]*	0.46	81	77 [±4.2]*	0.68	88	77 [±4.3]*	0.68
RE	□81.6 [±2.86]	67	58 [±7.3]*	100	69 [±6.7] *	0.30	83	60 [±7.7]*	0.88	100	75 [±6.0]	0.08	84	61 [±7.6]*	0.82
MH	□80.5 [±1.68]	64	62 [±3.4]*	66	65 [±4.0] *	0.58	60	63 [±3.6] *	0.79	68	63 [±3.7]*	0.83	66	64 [±3.7]*	0.70
EQ5D Index	#0.8 [±0.01]	0.80	0.73 [±0.04]	0.76	0.75 [±0.03]	0.91	0.73	0.73 [±0.04]	0.50	0.80	0.72 [±0.04]	0.71	0.80	0.70 [±0.04]*	0.35
EQ-5D VAS	#79.7 [±0.83]	75	73 [±3.0]*	79	74 [±2.8]*	0.76	75	70 [±3.4]**	0.98	75	71[±3.0]**	0.91	70	69 [±3.3]**	0.61

A1 = 3 months, A2 = 6 months, A3 = 9 months, A4 = 12 months, A5 = 15 months.

Data at A2–A5 were compared with data at A1 (linear mixed models for repeated observations).

□ Norm-based score = Norms for the general Swedish population (age group 60–64 years), Sullivan et al. 2002. # Norm-based score = UK population norms for the EQ-5D (age group 55–64 years), Kind et al. 1999.

PCS = Physical Component Summary Score, MCS = Mental Component Summary Score, PF = Physical Functioning, RP = Role Physical, BP = Bodily Pain, GH = General Health, VT = Vitality, SF = Social Functioning, RE = Role Emotional, MH = Mental Health, EQ-5D Index = EQ-5D index score, EQ-5D VAS = EQ-5D visual analog scale.

* p < 0.05, ** p < 0.01 = Statistically significant differences between norm-based scores and mean scores in the present study at A1–A5.

Table 2 also shows a comparison of norm-based scores with HRQOL scores. Mean scores for the Physical Component Summary and the four scales (Physical Functioning, Role Physical, Bodily Pain and General Health) of the SF-36 during follow-up were similar or higher compared with the norm-based scores. However, the Physical Component Summary Score was statistically significantly different (p < 0.05) only at A2. There were statistically significant lower mean scores compared with norm-based scores for the EQ-5D VAS at all assessments (A1–A5), but only at A5 for the EQ-5D Index (Table 2).

The mean scores for the Mental Component Summary as well as the four scales (Vitality, Social Functioning, Role Emotional and Mental Health) were lower than norm-based scores at all five assessments. These differences were statistically significant (p < 0.05), except for Role Emotional at A4.

Table 3 shows significant differences in SF-36 dimensions and scales between the two age groups (20–65 66–84 years). Respondents aged 20–65 years had statistically significantly higher scores, i.e. better HRQOL, on the Physical Component Summary and Physical Functioning scale over the complete follow-up period than respondents aged 66–84 years. Table 3 also shows that even though the younger age group had better physical HRQOL, they had worse mental HRQOL scores during the follow-up period, although only statistically significant at 3 months for the Mental Component Summary.

**Table 3:** HRQOL scores by age groups (n = 18 each) at 3 to 15 months (n = 36)

	**A1 Md**	**A1 M [SE]**	**p-value**	**A2 Md**	**A2 M [SE]**	**p-value**	**A3 Md**	**A3 M [SE]**	**p-value**	**A4 Md**	**A4 M [SE]**	**p-value**	**A5 Md**	**A5 M [SE]**	**p-value**
PCS															
20-65 years	55	52 [±2.3]	0.006**	56	55 [±2.7]	0.003**	50	50 [±2.1]	0.025*	52	50 [±2.7]	0.005**	54	52 [±2.7]	0.011*
66-84 years	40	41 [±2.5]		45	44 [±2.5]		42	42 [±2.6]		41	39 [±2.6]		45	42 [±2.4]	
MCS															
20-65 years	35	33 [±2.8]	0.007**	37	35 [±4.3]	0.036	36	35 [±4.0]	0.147	44	40 [±3.0]	0.117	40	36 [±3.9]	0.078
66-84 years	47	45 [±2.9]		47	47 [±2.3]		41	42 [±2.7]		49	46 [±3.0]		47	45 [±2.7]	
PF															
20-65 years	100	89 [±5.1]	<0.001***	100	94 [±2.7]	<0.001***	95	86 [±4.4]	0.012*	100	91 [±3.3]	0.001**	95	91 [±3.6]	0.006**
66-84 years	69	70 [±4.2]		75	74 [±4.9]		73	72 [±4.4]		75	69 [±4.7]		78	72 [±5.4]	
RP															
20-65 years	100	79 [±6.8]	0.021*	100	87 [±8.0]	0.107	100	68 [±10.3]	0.321	100	72 [±10.2]	0.174	75	71 [±10.2]	0.458
66-84 years	50	49 [±9.5]		75	67 [±9.2]		75	53 [±11.2]		50	53 [±10.0]		100	61 [±10.0]	
BP															
20-65 years	61	65 [±7.0]	0.649	72	71 [±7.1]	0.699	51	63 [±7.8]	0.440	57	66 [±6.8]	0.651	73	69 [±7.0]	0.271
66-84 years	41	60 [±6.8]		62	66 [±6.4]		51	57 [±6.3]		52	59 [±5.4]		57	60 [±4.9]	
GH															
20-65 years	62	62 [±4.8]	0.622	67	70 [±4.0]	0.303	77	68 [±5.2]	0.232	65	65 [±4.9]	0.070	70	70 [±5.1]	0.283
66-84 years	57	58 [±4.8]		67	61 [±4,8]		57	57 [±4.4]		62	57 [±4.0]		65	61 [±4.9]	
VT															
20-65 years	40	42 [±4.2]	0.013	55	51 [±6.2]	0.221	48	50 [±6.7]	0.726	55	54 [±5.0]	0.645	53	50 [±6.3]	0.341
66-84 years	65	58 [±4.2]		60	61 [±5.2]		50	54 [±4.4]		60	56 [±6.2]		60	59 [±4.2]	
SF															
20-65 years	75	68 [±6.4]	0.141	75	73 [±7.5]	0.262	83	64 [±7.0]	0.478	88	75 [±6.4]	0.646	75	72 [±6.6]	0.265
66-84 years	88	79 [±5.6]		100	83 [±5.3]		69	71 [±5.3]		75	78 [±5.6]		93	81 [±5.7]	
RE															
20-65 years	67	57 [±11.0]	0.878	100	60 [±11.8]	0.208	67	56 [±12.1]	0.694	100	76 [±8.5]	0.986	67	52 [±12.2]	0.286
66-84 years	67	59 [±10.1]		100	76 [±8.0]		83	63 [±10.0]		100	74 [±8.7]		100	68 [±9.5]	
MH															
20-65 years	50	53 [±4.0]	0.006	52	57 [±5.8]	0.061	56	57 [±6.4]	0.240	60	58 [±5.4]	0.193	62	60 [±6.1]	0.436
66-84 years	72	70 [±4.8]		76	71 [±5.4]		64	67 [±3.8]		76	68 [±4.9]		70	67 [±4.5]	
EQ-5D Index															
20-65 years	0.82	0.74 [±0.05]	0.591	0.73	0.73 [±0.05]	0.652	0.76	0.75 [±0.06]	0.392	0.80	0.80 [±0.05]	0.403	0.80	0.71 [±0.06]	0.254
66-84 years	0.73	0.72 [±0.05]		0.80	0.80 [±0.05]		0.73	0.71 [±0.04]		0.78	0.78 [±0.05]		0.76	0.70 [±0.06]	
EQ-5D VAS															
20-65 years	70	74 [±5.3]	0.774	70	70 [±4.6]	0.373	75	67 [±6.6]	0.973	75	73 [±4.3]	0.679	60	62 [±5.7]	0.115
66-84 years	75	72 [±3.5]		80	80 [±3.5]		75	72 [±3.0]		75	70 [±4.2]		80	75 [±3.3]	

Independent Samples Mann–Whitney *U* Test * p < 0.05, ** p < 0.01, *** p < 0.001, ns = not significant.

A1 = 3 months, A2 = 6 months, A3 = 9 months, A4 = 12 months, A5 = 15 months.

PCS = Physical Component Summary Score, MCS = Mental Component Summary Score, PF = Physical Functioning, RP = Role Physical, BP = Bodily Pain, GH = General Health, VT = Vitality, SF = Social Functioning, RE = Role Emotional, MH = Mental Health, EQ-5D Index = EQ-5D index score, EQ-5D VAS = EQ-5D visual analog scale.

Table 4 depicts comparisons between partners and children of the cancer patients with regard to the physical and mental scales during the study period. As shown, children scored statistically significantly higher on the Physical Component Summary than partners at all five assessments. Table 4 also shows that children had higher values than partners on the EQ-5D index, but differences were statistically significant only at 9, 12 and 15-month assessments (A3–A5). In summary, results showed that older age (Table 3) and being a partner (Table 4) had a negative influence on HRQOL, especially on the Physical Component Summary Score and Physical Functioning scale.

**Table 4:** **HRQOL scores by partners (n = 26) and children (n = 8**) **at 3 to 15 months (n = 36)**

	**A1 Md**	**A1 M [SE]**	**p-value**	**A2 Md**	**A2 M [SE]**	**p-value**	**A3 Md**	**A3 M [SE]**	**p-value**	**A4 Md**	**A4 M [SE]**	**p-value**	**A5 Md**	**A5 M [SE]**	**p-value**
PCS															
Partners	41	43 [±2.2]	0.015*	46	44 [±2.2]	0.006**	43	42 [±2.1]	0.019*	44	41 [±2.2]	<0.001***	48	44 [±2.1]	0.002**
Children	55	54 [±3.4]		57	58 [±1.8]		56	55 [±1.9]		56	56 [±7]		57	58 [±3.9]	
PF															
Partners	75	74 [±2.8]	0.002**	80	77 [±4.1]	0.001**	73	73 [±3.8]	0.007**	75	72 [±3.8]	#	80	75 [±4.3]	0.001**
Children	100	89 [±9.9]		100	99 [±1.3]		100	94 [±4.9]		100	100 [±0.0]		100	98 [±1.5]	
RP															
Partners	50	54 [±7.8]	0.181	75	66 [±8.4]	0.010*	50	53 [±9.2]	0.007**	63	57 [±8.6]	<0.001***	75	63 [±8.2]	0.001**
Children	100	84 [±8.1]		100	97 [±3.1]		100	84 [±12.4]		100	81 [±12.3]		100	82 [±14.1]	
BP															
Partners	41	56 [±5.7]	0.074	62	62 [±5.7]	0.175	51	55 [±4.5]	0.060	41	52 [4.9]	<0.007**	51	58 [±4.3]	0.004**
Children	92	82 [±8.0]		78	78 [±7.8]		92	81 [±8.4]		100	87 [±10.9]		100	89 [±8.4]	
GH															
Partners	57	59 [±4.2]	0.283	67	60 [±4.2]	0.205	62	56 [±3.2]	0.019*	60	56 [±3.8]	0.020*	67	61 [±4.0]	0.034*
Children	67	67 [±5.8]		70	72 [±2.7]		80	73 [±8.0]		80	78 [±7.0]		87	82 [±6.7]	
VT															
Partners	58	54 [±4.1]	0.148	55	59 [±4.3]	0.502	50	54 [±3.8]	0.413	60	55 [±5.0]	0.596	60	56 [±4.0]	0.567
Children	45	42 [±4.3]		58	50 [±10.8]		65	59 [±10.5]		58	62 [±5.5]		70	60 [±8.6]	
SF															
Partners	88	76 [±5.4]	0.383	100	84 [±4.6]	0.043	69	72 [±4.3]	0.804	75	78 [±4.5]	0.983	88	81 [±4.7]	0.226
Children	75	72 [±7.4]		56	61 [±11.4]		69	69 [±9.4]		88	77 [±10.1]		75	66 [±10.8]	
RE															
Partners	50	53 [±8.9]	0.222	78	75 [±7.0]	0.258	83	61 [±8.9]	0.850	100	74 [±7.2]	0.709	67	60 [±8.8]	0.729
Children	100	75 [±13.7]		67	54 [±17.7]		83	58 [±17.5]		100	83 [±8.9]		75	66 [±10.8]	
MH															
Partners	68	65 [±4.1]	0.103	68	69 [±4.5]	0.148	60	62 [±3.7]	0.881	68	65 [±4.0]	0.919	72	66 [±4.2]	0.749
Children	51	53 [±6.3]		46	54 [±10.0]		74	70 [±9.6]		73	65 [±7.8]		100	67 [±17.8]	
EQ-5D Index															
Partners	0.73	0.70 [±0.46]	0.068	0.76	0.76 [±0.04]	0.680	0.73	0.70 [±0.04]	0.011*	0.76	0.69 [±0.04]	0.002**	0.73	0.67 [±0.05]	0.001**
Children	0.85	0.85 [±0.07]		0.84	0.73 [±0.09]		0.92	0.89 [±0.05]		0.85	0.89 [±0.04]		0.85	0.86 [±0.03]	
EQ-5D VAS															
Partners	75	73 [±2.8]	0.415	80	76 [±2.8]	0.318	75	71 [±2.9]	0.365	75	70 [±3.5]	0.107	79	71 [±3.7]	0.292
Children	78	72 [±10.3]		67	65 [±8.7]		83	73 [±9.6]		78	81 [±3.8]		63	65 [±6.5]	

Independent Samples Mann–Whitney *U* Test * p <0.05, ** p <0.01, *** p <0.001.

A1 = 3 months, A2 = 6 months, A3 = 9 months, A4 = 12 months, A5 = 15 months.

PCS = Physical Component Summary Score, MCS = Mental Component Summary Score PF = Physical Functioning, RP = Role Physical, BP = Bodily Pain, GH = General Health, VT = Vitality, SF = Social Functioning, RE = Role Emotional, MH = Mental Health, EQ-5D Index = EQ-5D Index score, EQ-5D VAS = EQ-5D Visual analog scale.

# Not tested because values for children are constant (=100).

Nearly 56% of all partners were in the age range of 66–84 years, and 20% of the children were 20–65 years of age. There was a clear relationship between the partner variable and age variable (Rs = 0.6). The logistic regression analysis showed an age group effect on HRQOL for Physical Functioning at all five assessments: at A1 (p = 0.008, OR = 0.9), A2 (p = 0.007, OR = 0.8), A3 (p = 0.04, OR = 0.95), A4 (p = 0.004, OR = 0.9) and A5 (p = 0.01, OR = 0.9). At A1 age group explained differences in Vitality (p = 0.01, OR = 1.1), at A2 Role Physical (p = 0.02, OR = 1.1), and at A5 Role Emotional (p = 0.04, OR = 1.03). These results verify that age had the greatest effect on lower Physical Functioning scores, and can therefore be seen as a confounder to the explanation of the effect of cancer diagnosis on family members.

## Discussion

The present study revealed that family members of a person with lung or gastrointestinal cancer had poorer functioning on the mental dimension of HRQOL, compared with norm-based scores, indicating they felt worse than the general population [23,26]. Findings related to mental health-related quality of life measured by the SF-36 were considered clinically significant when 18 of 20 scale scores reflected a 10-point difference from norm-based scores. Standards for determining clinically significant change over time varies between different populations and is related to expectations of change. However, several studies have recommended 10 as an approximate cut-off representing a small change, 20 for a moderate change and 30 for a large change on scales of the SF-36 [28,29]. Our results on mental HRQOL, in terms of clinical relevance, highlight the importance of physicians and health care professionals providing support measures integrated into treatment and care.

Findings related to degree of change in the SF-36 physical dimension compared to the mental dimension were not consistently the same. The statistically significant difference in the EQ-5D VAS compared to the norm-based score represented an unequivocal pattern in all five assessments. However, the clinical significance of such findings was less clear, given that only three of the five assessments showed a difference in score of 10 points compared with the norm-based score. This result needs to be verified in large-scale studies in the future. This recommendation is supported by the review by Swore Fletcher and colleagues [16] that identified very few published studies about cancer’s impact on family caregivers’ physical health.

It is well-known that a cancer diagnosis is distressing not only for the sick person, but for close family as well. Previous research has shown a negative effect of the cancer diagnosis on the mental health dimension of HRQOL for family members with symptoms such as anxiety and depression [2,8,9,30,31]. Results reported by Persson and colleagues [11] are consistent with our finding that family members scored significantly lower on the mental dimension scales compared to the norm-based scores [11]. The literature reflects an interest in generating knowledge about how coping influences mental health. In a study by Sjölander and colleagues [32], findings showed that family members of a person with advanced lung or gastrointestinal cancer strive to prepare themselves mentally for the anticipated tragedy and use several different management strategies to cope with the menacing future. These results are in line with several previous studies [11,32,33]. However, there is a need for more knowledge about family members’ coping strategies and their influence on HRQOL [34].

This study identified three groups of family members vulnerable to illness. Older age had a negative effect on HRQOL compared with younger age. This was especially seen in the Physical Component Summary Score and Physical Functioning scale, with significant differences in all five assessments (Table 3). Earlier studies have shown that the Physical Functioning scale, which measures limitations in performing daily activities, has a strong association with aging [23,35]. Our results regarding poorer scores for physical HRQOL in older family members verify results of Kim and colleagues [13,36]. Analyses at item level in this study revealed that age explained the decreased HRQOL, especially for items that reflect more strenuous physical activities in the physical dimension. This result is consistent with the physical fragility in older age, which is well described in the literature [37-39]. This is in accordance with results of the logistic regression analysis in this study showing that age was one factor in assessing physical functioning at all five measurement times.

In contrast, younger family members had worse mental HRQOL scores during the follow-up period in our study. This was shown in the Mental Component Summary Score when it was compared with the norm-based score for the same age group in a population sample in a study by Sullivan and colleagues [23]. Worse mental health in younger persons with illness compared with older persons has also been shown previously [40]. In a review by Harden and colleagues [41], caregivers in late middle age (50–64 years) seemed to have more problems with mental health and psychological well-being than older caregivers [41]. Younger caregivers may experience more social and economic problems, resulting in increased anxiety and depression [42,43] as well as negative effects on their family life [44]. This was supported in our study, with younger persons’ mental health scores remaining low for the duration of the study.

The third group of family members identified as vulnerable in this study were partners of the patient, particularly with regard to their physical health. As their closest support, partners have a vital role in the care of the diagnosed person, [1,9,31,45,46]. In our study, partners had worse scores for the physical health dimension than did the children. However, age might have had an influence on this result, given that most partners (56%) were older (66–84 years), with worse scores on physical health possibly owing to physical fragility in older adults.

### Methodological considerations

The study population included relatively few family members and resulted in low power in detecting small changes over time and differences between subgroups in the population. This was the case, despite our recruitment of family members from two medium-sized hospitals and a rather long inclusion period (2007–2010). There were drop-outs both before the start of the study and during the study. The selection of family members was accomplished through assistance by staff (nurses and physicians) who initially asked the patients and secondly by the patients who asked their family member about participation. This procedure explains why there is no information about the reasons for drop-outs and demographic data of the initial 200 family members or the seven that dropped out right after consenting to participate. Despite several reminders provided during the data collection procedure to prevent drop-outs, the initial recruitment could have been done differently. The initial number who dropped out might have been reduced if the family members had been approached personally by the researcher or only one responsible nurse at each hospital.

Of the 57 family members entering the investigation, a total of 36 completed the study with participation in all five data collections. Analysis of drop-outs showed that the 21 family members who dropped out during the study were not significantly different in age, gender, education, occupation or relationship from those who completed the study. In addition, a strength of the data is that there was no difference in HRQOL or EQ5D at baseline between drop-outs after the start of the study and the study group who participated in all five data collections, which reduces the chance that the study was not representative. However, the large non-response rate makes uncertain the study’s generalisability, which must be kept in mind when interpreting the results.

Another reason for the low statistical power [47] was the authors’ decision to include only family members who filled in the questionnaires in all five data collections. The authors assessed it as better if the same individuals were included in the comparison over time than comparing groups of individuals in which the size decreased because of drop-outs. Seeing changes over time can result in more reliable data. Even though the power could have increased if all individuals were included, uncertainty would increase because there was no single group. The drop-outs during the study were explained by increased sickness in the patients, a factor that was out of our control during the year-long data collection. This reason for drop-outs has been previously described in the literature related to severe or advanced cancer patients [11,21]. Therefore, the performed statistical analyses were less powerful, and logistic regression analysis should be considered from a descriptive perspective. In addition, the findings of the logistic regression analysis of type of relation on HRQOL were not included in the results because of a large difference in group size (partners, n = 28 and children, n = 8). The ability to predict values for the group of children was relatively low with percentage correct 50–62% compared with the analysis of partners (percentage correct 83%) and age (percentage correct 85%).

When a large number of comparisons are statistically examined, there is a risk of mass significance. With an alpha-level of .05, the risk for random significance is 1 in 20. One way to diminish the risk of mass significance is to lower the alpha level. Procedures used to calculate a new alpha level are Bonferroni’s test and the Dunn-Sidak correction [47]. However, in this study the authors determined that these tests would be too conservative and might increase the possibility of a type II statistical error. For this reason, weak or solitary significances should be considered with some scepticism. Furthermore, the collected data are ordinal-level and not equidistant, and thus there is a risk of bias in evaluating the size of changes [48] owing to the uneven distribution of such changes. Conclusions related to our results are therefore based on the patterns of significant differences that were in the same direction without deviant values. Linear mixed models can be used to describe nonlinear relationships across time in a longitudinal dataset with multiple missing data points. The strengths of the mixed models are the ability to accommodate missing data points and the ability to model nonlinear, individual characteristics. The mixed model emphasises patterns of change and individual differences and assumes not a normal distribution but rather systematic change.

The proportion of men in the study group of family members was low, which might be explained by the selection method. Both sexes were represented in nurses (females) and physicians (males) who asked about participation. However, 26 of the 36 patients who asked one of their family members to participate were males (72%), and 20 of them (77%) chose a female partner. Six of the males chose a grown child, and five of those were daughters. Cancer incidence and mortality in Europe predominately involves men with lung or upper gastrointestinal tract cancer [49]. These cancer diagnoses are the most common worldwide among men and constitute 42% of new cases and 48% of total cancer deaths. Given that a diagnosis of lung or upper gastrointestinal tract cancer is more common among men, it seems reasonable that our study group was predominately male cancer patients and female family members.

The longitudinal design [50] was appropriate for studying the dynamics of the variables over time given that cancer patients’ serious illness progresses rapidly and may affect the family members [50]. The 1-year follow-up period was considered appropriate in respect to investigating outcomes for family members dealing with the mortality of an advanced cancer patient.

## Conclusions

There were no statistically significant changes in physical or mental HRQOL in the study group over the one-year follow-up. The family members had poorer mental HRQOL scores throughout the period as measured by the SF-36 compared with the norm-based scores. In addition, mean scores for the EQ-5D VAS were statistically significantly lower at all assessments compared with norm-based scores. The results suggest that older family members of cancer patients are at higher risk for decreased physical HRQOL, especially if they are the partner. Younger family members are more vulnerable to decreased mental HRQOL. Older family members seem to cope with the situation more effectively than younger persons who show decreased mental HRQOL. This indicates that support programmes for younger family members need to focus more on emotional aspects. However, there is a need for larger-scale research before conclusions can be drawn regarding interventions individually tailored for family members vulnerable to illness.

## Competing interests

The authors declare that they have no competing interests.

## Authors’ contributions

CS, JJ, JM and GA designed the study, CS and JJ collected the data, CS and BR in collaboration with JM and GA performed the statistical analyses, CS, BR, JJ, JM and GA were responsible for the manuscript preparation. All authors read and approved the final manuscript.
